# Characterization of the complete chloroplast genome of *Astragalus galactites* (Fabaceae)

**DOI:** 10.1080/23802359.2021.1993105

**Published:** 2021-10-23

**Authors:** Xiaodong Ding, Chaopan Zhang, Yangqiong Gao, Zhanlin Bei, XingFu Yan

**Affiliations:** Key Laboratory of Ecological Protection of Agro-pastoral Ecotones in the Yellow River Basin National Ethnic Affairs Commission of the People’s Republic of China, School of Biological Science & Engineering, North Minzu University, Yinchuan, Ningxia, China

**Keywords:** Fabaceae, *Astragalus galactites*, chloroplast genome, phylogenetic analysis

## Abstract

*Astragalus galactites* is a medicinal plant. The total plastome length of *A. galactites* is 126,117 bp. It contains a large single-copy region of 69,805 bp, two inverted repeat regions of 20,638 bp, and a small single-copy region of 15,036 bp. The cp genome contains 110 complete genes, including 75 protein-coding genes (75 PCGs), 4 ribosomal RNA genes (4 rRNAs), and 30 tRNA genes (30 tRNAs). The overall GC content of cp DNA is 33.9%, the corresponding values of the LSC, SSC, and IR regions are 33.0%, 30.4%, and 43.3% respectively. The phylogenetic tree shows that *A. galactites* has the closest relationship with *A. laxmannii*.

*Astragalus galactites* Pall. (Ledebour 1842), a desert psammophyte belongs to the family Fabaceae. It distributes in the northern part of China, Mongolia, and Siberia. The root can be used as a traditional Chinese medicine to consolidate the exterior and induce diuresis to reduce edema. (Zhong et al. 2012; Yang et al. 2010). This fact is being increasingly substantiated by pharmacological studies showing that it can increase telomerase activity and has antioxidant and diuretic effects (Anon 2003; Zhao et al. 2011). Meanwhile, a systematic study of this species would have significant implications for understanding the origin and evolution of the genus *Astragalus* and local flora. However, the chloroplast genome of *A. galactites* has not been reported. In this study, the complete chloroplast genome of *A. galactites* has been assembled in order to lay a foundation for further research.

Fresh leaves of *A. galactites* were collected from Tongxin (Wuzhong, Ningxia, China; coordinates: 106.4904E, 37.1414 N) and dried with silica gel. The DNA and voucher specimen was stored in the Herbarium of North Minzu University (NMU) with the number is zlnmu2021002 (Lei Zhang: zhangsanshi-0319@163.com; Yuqing Wei: weiyuqing@126.com). The total genomic DNA was extracted with the modified CTAB method (Doyle and Doyle 1987) and a 350-bp library was constructed. This library was sequenced on the Illumina NovaSeq 6000 system with 150 bp paired-end reads. We obtained 10 million high-quality pair-end reads for *A. galactites*. After removing the adapters, the remaining reads were used to assemble the complete chloroplast genome by GetOrganelle pipeline v1.6.3a (Jin et al. 2020). The complete chloroplasts genome sequence of *A. gummifer* was used as a reference. Plann v1.1 (Huang and CronK 2015) and Geneious v11.0.3 (Kearse et al. 2012) were used to annotate the chloroplasts genome and correct the annotation.

The total plastome length of *A. galactites* (MZ504977) is 126,117 bp. It exhibits a typical quadripartite structural organization, consisting of a large single copy (LSC) region of 69,805 bp, two inverted repeats (IR) regions of 20,638 bp, and a small single copy (SSC) region of 15,036 bp. The cp genome contains 110 complete genes, including 75 protein-coding genes (75 PCGs), 4 ribosomal RNA genes (4 rRNAs), and 30 tRNA genes (30 tRNAs). The overall GC content of cp DNA is 33.9%. The corresponding values of the LSC, SSC, and IR regions are 33.0%, 30.4%, and 43.3%.

To further clarify the phylogenetic position of *Astragalus galactites*, the plastome of 10 representatives *Astragalus* species were obtained from NCBI to reconstruct the plastome phylogeny, with *Oxytropis bicolor* and *O. arctobia* being an outgroup. All the sequences were aligned by using MAFFT v.7.313 (Katoh and Standley 2013) and the maximum likelihood of phylogenetic analyses was conducted through RAxML v.8.2.11 (Stamatakis 2014) under the GTRCAT model with 1000 bootstrap replicates. The phylogenetic tree shows that all *Astragalus* pecies were divided into two subclades (Figure 1). *A. galactites* has the closest relationship with *A. laxmannii*.

**Figure 1.**
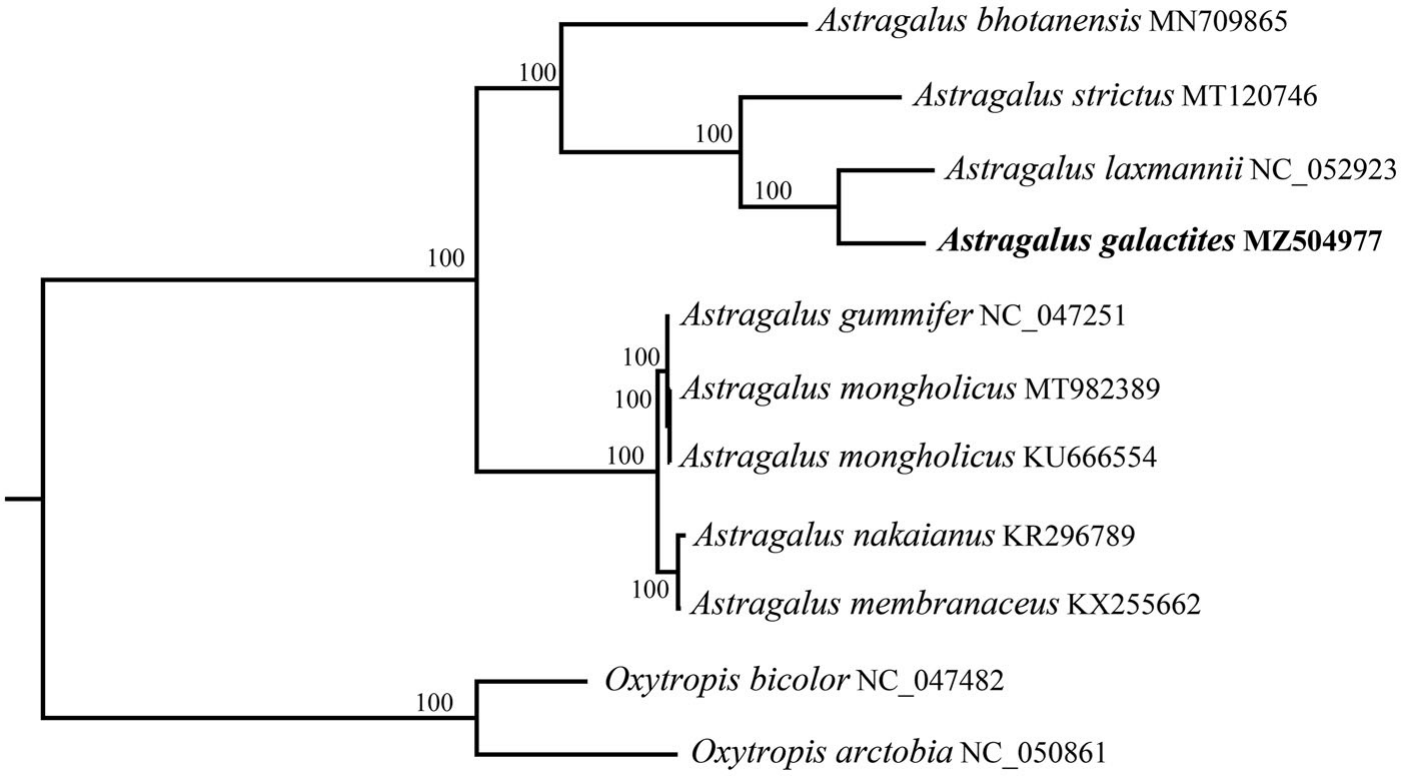
Phylogenetic relationships of *Astragalus* pecies using whole chloroplast genome. GenBank accession numbers: *Astragalus bhotanensis* (MN709865), *Astragalus strictus* (MT120746), *Astragalus laxmannii* (NC_052923), *Astragalus galactites* (MZ504977), *Astragalus gummifer* (NC_047251), *Astragalus mongholicus* (*MT982389*), *Astragalus mongholicus* (KU666554), *Astragalus nakaianus* (KR296789), *Astragalus membranaceus* (KX255662), *Oxytropis bicolor* (NC_047482), *Oxytropis arctobia* (NC_050861).

## Data Availability

The data that support the findings of this study are openly available in GenBank of NCBI at https://www.ncbi.nlm.nih.gov, reference number MZ504977. The associated BioProject, SRA, and Bio-Sample numbers are PRJNA746116 SRA: SRS2859862 and SAMN20181703, respectively.
